# Reflections on the Use of an Invertebrate Chordate Model System for Studies of Gut Microbial Immune Interactions

**DOI:** 10.3389/fimmu.2021.642687

**Published:** 2021-02-25

**Authors:** Assunta Liberti, Ojas Natarajan, Celine Grace F. Atkinson, Paolo Sordino, Larry J. Dishaw

**Affiliations:** ^1^ Biology and Evolution of Marine Organisms (BEOM), Stazione Zoologica Anton Dohrn, Naples, Italy; ^2^ Morsani College of Medicine, Department of Pediatrics, University of South Florida, Tampa, FL, United States; ^3^ Division of Molecular Genetics, Children’s Research Institute, St. Petersburg, FL, United States; ^4^ Department of Cell Biology, Microbiology, and Molecular Biology, University of South Florida, Tampa, FL, United States

**Keywords:** *Ciona robusta*, *Ciona intestinalis* type A, invertebrate model, mucosal immunity, innate immunity, gut-microbial interactions, gut microbiota, multi-omics approaches

## Abstract

The functional ecology of the gastrointestinal tract impacts host physiology, and its dysregulation is at the center of various diseases. The immune system, and specifically innate immunity, plays a fundamental role in modulating the interface of host and microbes in the gut. While humans remain a primary focus of research in this field, the use of diverse model systems help inform us of the fundamental principles legislating homeostasis in the gut. Invertebrates, which lack vertebrate-style adaptive immunity, can help define conserved features of innate immunity that shape the gut ecosystem. In this context, we previously proposed the use of a marine invertebrate, the protochordate *Ciona robusta*, as a novel tractable model system for studies of host-microbiome interactions. Significant progress, reviewed herein, has been made to fulfill that vision. We examine and review discoveries from *Ciona* that include roles for a secreted immune effector interacting with elements of the microbiota, as well as chitin-rich mucus lining the gut epithelium, the gut-associated microbiome of adults, and the establishment of a large catalog of cultured isolates with which juveniles can be colonized. Also discussed is the establishment of methods to rear the animals germ-free, an essential technology for dissecting the symbiotic interactions at play. As the foundation is now set to extend these studies into the future, broadening our comprehension of how host effectors shape the ecology of these microbial communities in ways that establish and maintain homeostasis will require full utilization of “multi-omics” approaches to merge computational sciences, modeling, and experimental biology in hypothesis-driven investigations.

## Introduction

The gut environment includes a dynamic community of microorganisms, consisting mainly of bacteria, but also Archaea, viruses, fungi, protozoans, and occasionally, helminthic worms. In the past few decades, the beneficial effects of these microbes on animal health have become widely recognized, influencing host physiology at different levels, i.e., immune system and gut, as well as other organs, development, and metabolic and neurobehavioral functions (1–4). Dysregulation of homeostasis, in terms of both microbial composition and host capability to regulate interactions with microbes, is most often correlated with intestinal pathologies (5–9).

The immune system, and more specifically innate immunity, plays a fundamental role in modulating the interface of host and microorganisms in the gut. It is equally true that microorganisms shape host immunity (10–12) and that host immunity shapes gut microbial communities (13, 14). The innate immune system may have evolved not only for defense but also driven by a necessity to recognize and tolerate complex communities of beneficial microbes, representing a form of ecosystem management that modulates their composition, diversity, and localization (2, 15, 16). To decipher the relationships between microbes and host physiology and/or diseases, this field of study has been primarily focused on humans. However, fundamental questions remain, necessitating the use of diverse model systems, including those that are “simpler” (17). These model systems can help inform us of the central principles legislating homeostasis in the gut and, specifically, will help refine our recognition of the role(s) of innate immunity in governing the complex gut ecosystem.

**Figure 1:**
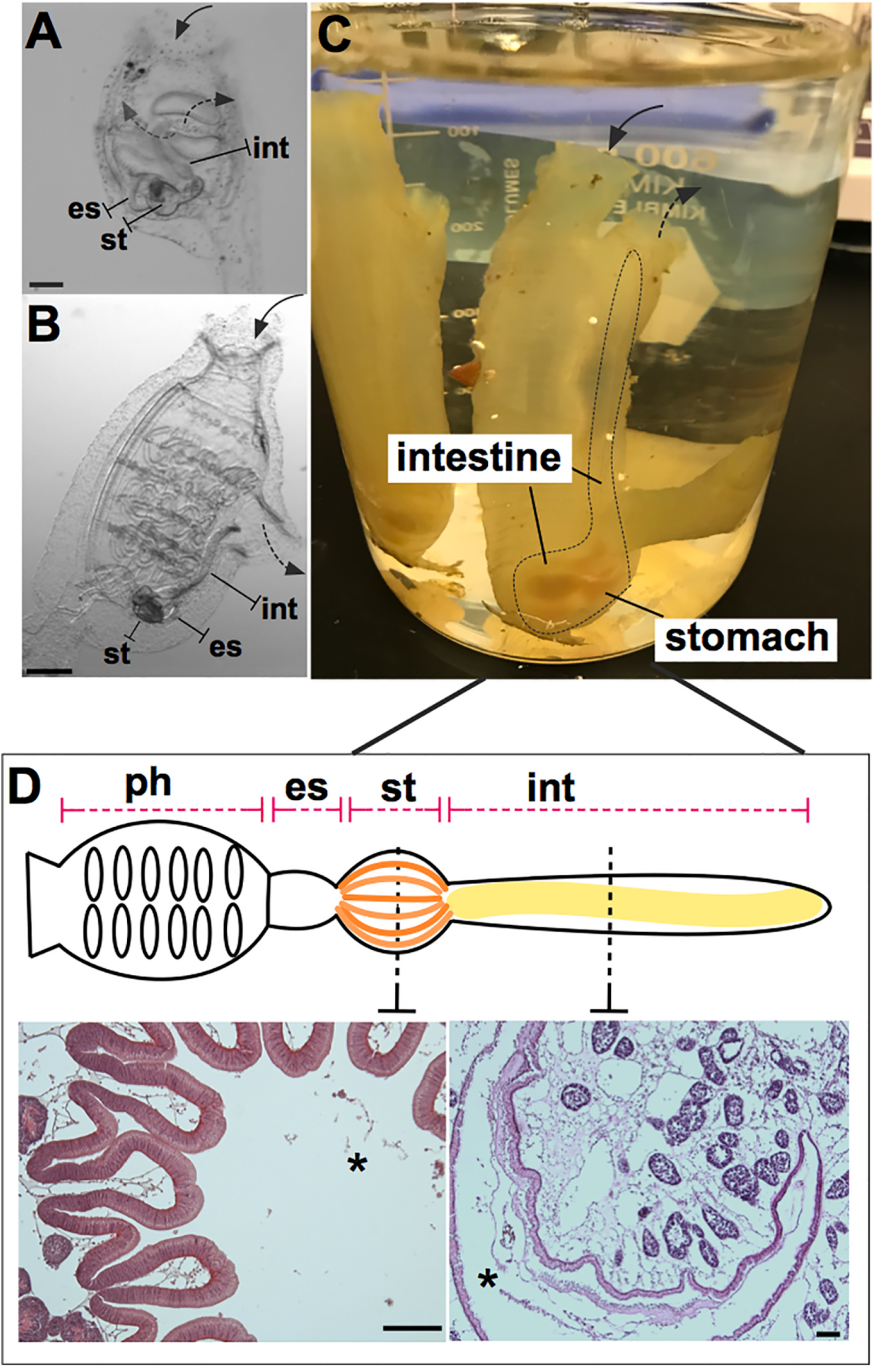
*Ciona robusta* as a model organism for studying gut-microbial interactions. *Ciona* is a solitary invertebrate chordate that typically grows in close proximity on suitable substrates. Embryonic development results in a larval stage that attaches to a substrate and undergoes metamorphosis to achieve the adult phenotype. **(A)** At stage 4 of metamorphosis, translucent juveniles open their siphons and begin to filter seawater, where they initiate feeding; the gut is first exposed to and colonized by microorganisms. **(B)** The stage 8 juvenile has completed metamorphosis and exhibits the anatomical structures of a young adult. **(C)** Field-harvested full adult, with digestive tract emphasized by dotted line; water flow (first entering the pharynx) depicted by arrows. **(D)** Graphical (linear) representation of Ciona gut includes the pharynx, esophagus, stomach, and intestine. Hematoxylin/eosin staining of the stomach (left) and intestine (right) is shown, highlighting the folded organization of the stomach epithelium and the smooth layer of the intestinal epithelium. Ph, pharynx; es, esophagus; st, stomach; int, intestine; solid arrow, oral siphon; dotted arrow, atrial siphon; arrows direction depicts water flow. Asterisk, gut lumen. Scale bars: **(A)**, 100 µm; **(B)**, 500 µm; **(D)** 100 µm.

It was argued previously (18) that the invertebrate chordate, *Ciona robusta*, could serve as a novel, informative, model system for studies of host-microbiome interactions. *Ciona* has long been a well-established model for investigating animal development (19–22) and immune defense (23) due to its experimental tractability and its phylogenetic position relative to vertebrates (24). These prior studies were focused on what we now recognize as *C. robusta* but were published as *C. intestinalis* (Type A). Further, with anatomic features of a digestive tract that are easy to identify and dissect, its reliance on innate immunity, along with husbandry approaches to rear thousands of transparent filter-feeding juveniles, reveals *Ciona* as a suitable organism for studying gut homeostasis (**Figures 1A–C**). Further, this model helps reveal the essential roles of innate immunity in shaping the dynamic interaction between host and microbiome, especially given the unique vantage point of a siphoning filter-feeder that moves large volumes of microbe-rich seawater across its mucosal epithelium. Significant progress, reviewed herein, has been made to fulfill this vision.

Here, we will recapitulate recent efforts to characterize the *Ciona* gut environment, including the presence of a gut epithelium layered with chitin-rich mucus and roles for a secreted immune effector family, namely the immunoglobulin (Ig)-like variable region-containing chitin-binding proteins (VCBPs), interacting with distinct elements of the microbiome. We will review the gut microbiome of adults, which includes abundant bacteriophages, and the establishment of a large catalog of cultured isolates from which cultured juveniles can be colonized for experimental manipulation and study. And finally, we will discuss the establishment of methods to rear short-term germ-free animals, an essential requirement for the interrogation of each member of the symbiotic dialogue existing within the gut microbiome. As the foundation is now set, future studies can be extended further to include large-scale analyses of host–microbe interactions. Such studies will require the use of “multi-omics” approaches, thus merging computational sciences, modeling, and experimental biology in hypothesis-driven investigations, broadening our comprehension of how host effectors shape the ecology of these microbial communities in ways that establish and maintain homeostasis, while this equilibrium is challenged by diverse environmental stressors.

## Components of *Ciona* Gut Environment

**Figure 2:**
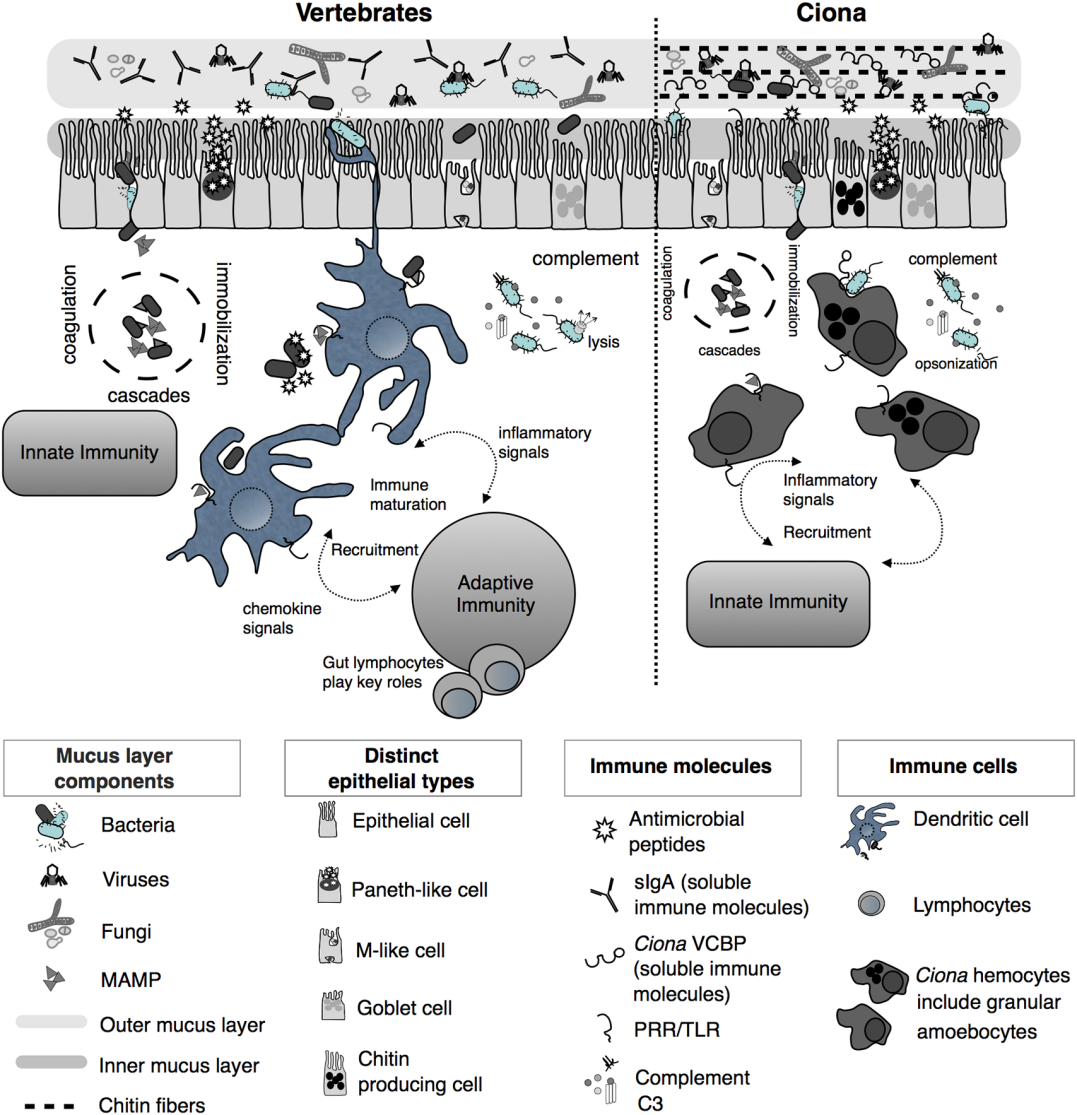
Simplified illustration of mucosal immunity emphasizing barrier defense strategies of vertebrates and *Ciona* (Reprinted and updated from [18)]. Gut epithelium represents a primary barrier of defense, governed by innate immune phenomena characterized by the secretion of mucus (that organizes as inner, compact, and firmly attached inner layer and a looser outer layer), antimicrobial peptides (AMPs), and soluble immune molecules. The secreted outer mucus layers are often colonized by diverse microorganisms, including bacteria, viruses, and fungi. In vertebrates, on the basolateral surface of the epithelium, host innate immunity consists of various proteolytic-coagulation cascades for wound-healing and microbial trapping, as well as complement defense pathways. Phagocytic cells, i.e., dendritic cells (DCs), as well as other cell types, populate this area. DCs sample luminal antigens and present them to the adaptive immune system, which includes gut-specific lymphocytes of both T and B cell lineages, thus triggering the maturation of immunity and the recruitment of additional cell types. In *Ciona*, a more simplified system includes an epithelial barrier, consisting of distinct epithelial lineages and the secretion of immune mediators, including AMPs and soluble immune molecules such as immunoglobulin (Ig)-like variable region-containing chitin-binding domains (VCBPs), into the lumen. The epithelium-associated mucus also consists of chitin fibers that run parallel to the epithelium and that are recognized and bound by VCBPs [*via* its chitin-binding domain (CBD)]; with opposing domain structures, free VCBP-C in the lumen can bind both bacteria and fungi. In the basolateral side, a distinct population of hemocytes, i.e., granular amoebocytes, resides in the laminar connective tissue. As in vertebrates, immunological competence in this area is mediated by coagulation/immobilization cascades and microbial trapping, complement defenses, antigenic sampling *via* pattern recognition receptors (PRRs), secretion of pro-inflammatory signaling, and recruitment of specialized hemocytes. However, as opposed to the vertebrate gut, *Ciona* relies on just innate immune mechanisms, without coupling them to the more specialized adaptive immune system.

Host defense systems serve bifunctional roles in protecting host tissues from pathogenic infection while also supporting the growth of specific communities of microbes (25–27). This dichotomy serves to ensure proper nutritional sustenance for the host, while training host immunological systems to handle the load of ingested microbes, resisting those that are pathogenic or infectious. Phylogenetically, the role of the innate immune system in maintaining microbial communities in the gut has ancient origins (25). To ensure this dual function of the immune system, the whole gut has evolved distinct anatomical, morphological, and functional characteristics. Indeed, as a filter-feeding invertebrate most closely related to vertebrates, *Ciona* represents an attractive and useful model for research into understanding the evolution and diversification of the digestive systems in chordates (28, 29). Like other ascidians, *Ciona* possesses a U-shaped alimentary canal that includes a pharynx, esophagus, stomach, and intestine (**Figure 1D**). The stomach epithelium is folded and organized with many ciliated ridges and grooves, and characterized by four cell types: ciliated mucus cells, gland cells, vacuolated cells, and undifferentiated cells. The intestinal epithelium, instead, appears as a smooth layer, with absorptive cells and narrow mucous cells (30–33). Thus, the highly developed and compartmentalized gut of *Ciona* morphologically resembles that of more recently diverged chordates (31, 33). The specific functions of each compartment have yet to be well defined. Beyond the anatomical organization, an important function of homeostasis in the gut is the establishment of a mucosal environment where immune cells, epithelial-mesenchymal cells, and commensal microbes are combined to promote the formation and existence of the gut ecosystem (34). Epithelial barriers and associated innate immune functions are phylogenetically ancient and have evolved diverse languages to help sustain a stable dialogue with adherent microorganisms (25). In *Ciona*, various components of the gut mucosal surface have been identified (**Figure 2**) and are described below.

### Mucosal Surface Barrier of *Ciona* Gut

The first barrier that ensures gut homeostasis is made by host components and is comprised of a mucus layer and immunomodulators. In mammals, these components have been and continue to be thoroughly investigated. The composition and organization of the mucus layers along the different compartments of the digestive tract have been described (35–38). In addition, diverse secreted immune molecules have been identified and functionally characterized, e.g., immunoglobulin A (IgA), RegIII*γ*; antimicrobial peptides (AMPs) (**Figure 2**) (39–45).

#### Mucus Layer

**Figure 3:**
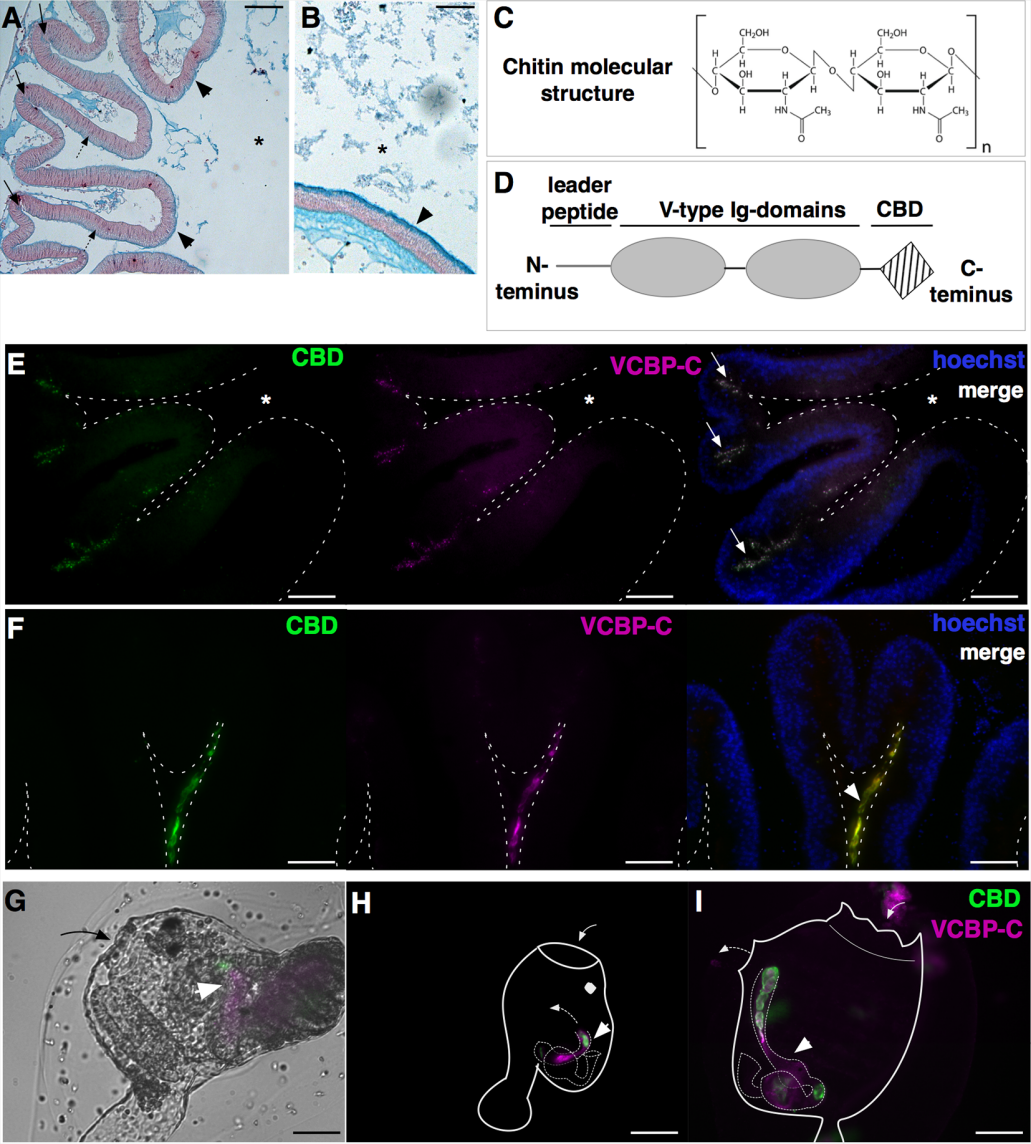
Components of *Ciona* gut mucosal surface. Gut sections stained with Alcian blue, which stains acidic mucopolysaccharides, reveals **(A)** a thin and dense mucus layer (arrowheads) and glycoprotein-rich vesicles within cells localized in the crypts (arrows) and spread along the apical side of the epithelium, in the stomach (dotted arrows), and **(B)** a thicker mucus layer along the mid- to distal gut (arrowheads). **(C)** Chitin is a polymer of β(1-4)-linked N-acetyl-d-glucosamine residues. **(D)** VCBP protein structure, consisting of a leader peptide, two tandem N-terminal V-type immunoglobulin domains, and a C-terminus chitin-binding domain (CBD). **(E–I)** Double immunostaining with recombinant IgG1-Fc-CBD-C probe (validated in (46, 47) (green) recognizing chitin molecules and VCBP-C detected with a rabbit polyclonal antibody [validated in (48, 49)] (magenta). **(E, F)** Section of stomach epithelium shows colocalization of chitin molecules and VCBP-C proteins in both granules of cells localized in stomach crypts (**E**, arrows) and in the mucus lining the stomach epithelium (**F**, arrowhead). During metamorphosis, chitin fibers and VCBP-C colocalized in **(G)** the developing gut of juveniles at the early rotation stage (arrowhead), **(H)** the intestine and **(I)** the stomach and the intestine of juveniles at stage 4 and 8 of metamorphosis, respectively (arrowhead). In **(G, I)**, solid arrow and dotted arrow depict oral siphon and atrial siphon, respectively; in **(H, I)**, arrow directions depict water flow. Asterisk, gut lumen. Scale bar: **(A)** 100 µm; **(B, E-I)** 50 µm.

In *Ciona*, the mucus lining the gut epithelium of adult individuals consists primarily of acidic mucopolysaccharides and endogenously produced chitin (46). The layers immediately adjacent to the epithelium are more densely arranged or rigid and resemble the intestinal glycocalyx present in many metazoans. The outer layers of mucus are organized as strands that run parallel to the surface epithelium; its arrangement appears thinner in the stomach and thicker, and more loosely arranged, as one travels from the midgut to the distal gut area where microbes are typically most abundant (**Figures 3A, B**) (46). This chitin-rich mucus of *Ciona* also serves important roles in confining ingested food particulates and microbes to the luminal space, maintaining the ciliated epithelial surface free of microbes (46, 50). The layering of mucus and the formation of defined microbe-rich and microbe-poor boundaries have also been observed in the intestines of mammals (37, 38).

The presence of the main constituents of mammalian mucus layers, i.e., glycoproteins such as mucins (MUCs), has been identified in nearly all metazoans (51); however, mucus chemistry and organization can differ considerably, e.g., in mammals, multiple MUC glycoproteins exist, with unique distributions in each mucosal tissue type. In many arthropods, the midgut intestine includes mucus rich in glycoproteins known as peritrophins; the resulting “peritrophic matrix” includes chitin fibers that reinforce the glycoprotein-rich MUCs (52–55). In the *C. robusta* genome, several MUC-encoding genes have been identified (51). Mass spectrometry-based proteomic analyses of mucus isolated from *Ciona* reveal the presence of matrix components that include a large mosaic protein (2880 amino acids) with 30 domains of 13 types, whose overall arrangement is conserved with human gel forming MUCs (GFMs) (50). The localization, organization, and function of this newly identified Ci-GFM1 remain to be investigated.

While the *Ciona* mucus is particularly enriched in chitin fibers, a biopolymer of N-acetylglucosamine (**Figure 3C**), its function in the gut has been primarily studied in arthropods (52, 56). The observation of chitin-rich mucus in non-mammalian vertebrates, such as fishes and amphibians (57), suggests a broader phylogenetic distribution than considered previously. In *Ciona*, chitin-rich mucus can form a thick ribbon-like structure, as seen lining the stomach epithelial crypts (46); these include cells containing chitin-rich granules (**Figures 3E, F**) (47). As described above, the rest of the intestinal compartment is also lined with this reinforced mucus (46). The endogenous production of chitin fibrils, specifically within the digestive tract, has been demonstrated by whole mount *in situ* localization of chitin synthase mRNA, the enzyme producing chitin, in the stomach and intestine of *Ciona* juveniles (46, 50). Whole mount immunofluorescence detection of chitin fibers is also revealed at various stages of *Ciona* metamorphosis (46). Prior to the onset of feeding, chitin localizes within the tube-shaped structure of the developing intestine in *Ciona* juveniles, and then fills the gut lumen in the form of chitin-rich pellets observable along the digestive tract later in metamorphosis, as juveniles start to filter seawater (**Figures 3G–I**) (46). It has been hypothesized that the pellets help establish the cylindrical lumen as it extends from the pharynx through to the anus, while later in development, chitin-rich mucus begins to line the intestinal epithelium (46). However, further studies are required to test this hypothesis.

#### Immune Molecules

The gut is a mucosal environment. Animals secrete soluble immune effectors into the lumen of mucosal tissues; effectors with Ig domains, such as IgA in mammals, are one example. Ig domains have evolved into diverse roles, with antigen binding and immunity serving cornerstone functionalities (58). Within the *Ciona* gut environment, secreted immune molecules containing Ig domains, namely VCBPs, have been described (46, 48, 49). These molecules are characterized by two tandem variable (V)-type Ig-like domains at the N-terminus and a single chitin-binding domain (CBD) at the C-terminus (**Figure 3D**). Genome organization and molecular structure have been resolved and reviewed previously (59).

In *Ciona*, four unlinked VCBPs have been identified, namely VCBP-A, VCBP-B, VCBP-C, and VCBP-D, with the latter lacking the CBD at the C-terminus (48, 60, 61). VCBP-A, -B, and -C expression patterns have been described in the digestive tract and in the blood cells (48, 49). Specifically, during metamorphosis, VCBP-A and VCBP-C present distinct spatio-temporal expression patterns in the developing gut: VCBP-A is localized in the developing stomach and VCBP-C is expressed, at first, in the primordium of the intestine, where chitin-rich fibrils are detected (46), and then also in the stomach (49, 59). In the adult, the expression of VCBP-A in the stomach is replaced by VCBP-B, whose expression is observed in scattered cells along stomach epithelium (48, 49), and VCBP-C is still localized within cells in the crypts of stomach villi and in the distal part of the intestine (**Figures 3E–I**) (48, 49). VCBP-C molecules are also observed in the gut lumen, either in contact with the epithelium or within the chitin-rich mucus layer, co-localizing with both chitin-rich fibrils and bacteria (**Figures 3E, F** and **6A, B**) (46, 48). VCBP-A and VCBP-C expression is also described in blood cells, and in some instances, amoebocytes expressing VCBP-A are found associated with the basement membrane of the stomach epithelium (49). Moreover, VCBP-C acts as an opsonin, increasing the phagocytic activity of granular amoebocytes that are pre-incubated with either affinity purified VCBP-C, i.e., native, or with recombinant VCBP-C (48). Proteomic analysis of chitin-rich mucus further confirms the main presence of VCBP-C, together with the detection of a putative, secreted, pore-forming protein of the membrane-attack complex/perforin (MACPF) family, namely Ci-MACPF1 (50). The function of this latter molecule, in *Ciona*, is not yet described, although cytolytic activities have been demonstrated in other organisms (63). VCBP genes have also been found in another protochordate, the cephalochordate amphioxus, *Branchiostoma floridae* (60, 61). The finding that secreted Ig-containing immune effectors are not restricted to vertebrates suggests that these types of immune molecules present selective advantages within the gut of diverse chordate lineages (58, 64).

Additional innate immune molecules encoded in the *Ciona* genome include Toll-like receptors (TLRs), which are also expressed in the gut environment (**Figure 2**) (29, 65, 66). Originally, only two TLRs, namely TLR1 and TLR2, were identified and structurally characterized (65); however, more recent computational efforts using improved genome assemblies identify a third putative TLR (67). The number of TLRs in the genomes of animals appears to vary widely; for example, between two other deuterostome invertebrates, *B. floridae* and *Strongylocentrotus purpuratus*, 72 and 253 TLR genes, respectively, have been described (68, 69). The finding that *Ciona* possesses a significantly reduced number of TLRs makes it an attractive model for functional studies of this essential innate effector family (29). The TLRs belong to the broad family of pattern recognition receptors (PRR), whose signaling is critical in coordinating immune responses and protection against pathogens (70, 71). However, PRRs are also likely interacting often with commensal microbes, suggesting important roles in mediating cross-talk between symbionts and their animal hosts (72). In mammals, TLRs are expressed in macrophages and dendritic cells where they act as pro-inflammatory molecules during infections (73, 74), but they are also expressed in intestinal epithelial cells, generally in a polarized fashion restricted to the basolateral surface or within endosomes of the cells (75, 76). TLRs are important for the maintenance of spatial segregation of microbes in the gut lumen (40, 77) and for shaping the microbial community structure (78, 79). Similarly, the expression of both TLRs in *Ciona* occurs widely throughout the digestive tract, both in the stomach and in the intestine, as well as in hemocytes (65). *In vitro* analyses also confirm that they are localized on both plasma membranes and in late endosomes, without demonstrating a specific localization to precise cellular compartments as observed in mammals, suggesting in *Ciona*, a promiscuity of function or microbial recognition (65).

### Microbiota of *Ciona* Gut

The microbial communities residing in the gut are generally referred to as the gut microbiota and while they represent exogenous elements, their integral role to animal physiology is now widely recognized. The metabolic output of the gut microbiota is roughly equivalent to an organ, and this extended phenotype has been referred to as a “forgotten organ” (80). While the microbiome includes primarily beneficial or commensal organisms, on occasion some pathogens visit or current inhabitants become pathogenic or harmful after shifts in microbial community structure, i.e., pathobiont or opportunist (81). Therefore, an important consideration in studies of host-microbiome interactions is knowledge of what are the expected or typical compositions of microbial communities residing within the gastrointestinal tract. For example, does the temporal persistence of specific taxa suggest a functional “core” microbiota of physiological importance, required for optimal animal health or survival? These impressions are important starting points in designing studies that focus on the ecology of the system, such as *in vitro* polymicrobial interactions influenced by biotic or abiotic factors, and further determining if these outcomes influence host physiology. Dissecting the interplay among diverse members of these complex microbial communities is also relevant in other fields of research, e.g., phylosymbiosis, which aims to understand if reciprocal interactions within a holobiont shapes phylogenies (82, 83).

**Figure 4:**
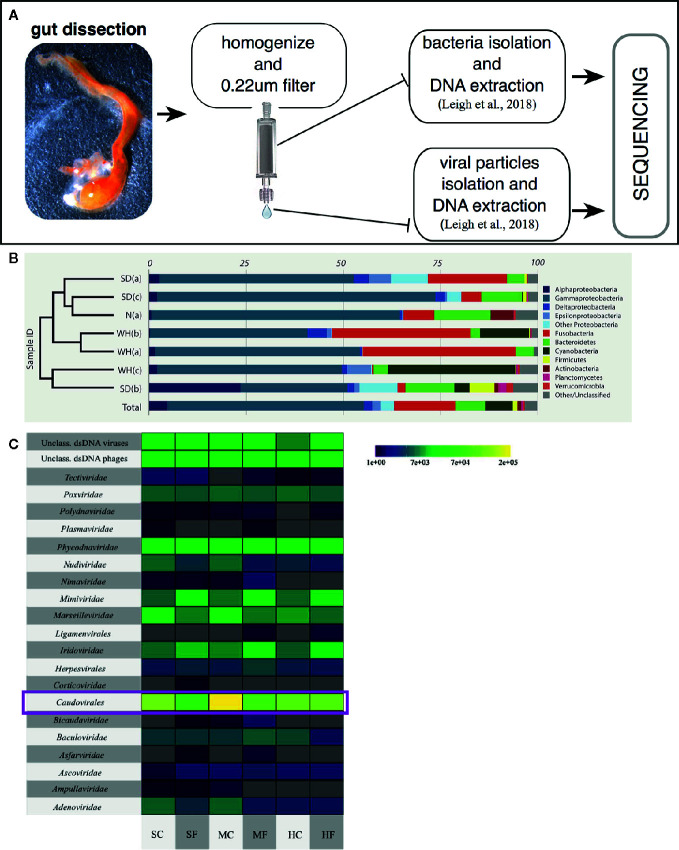
Characterizing the *Ciona* gut microbiota. **(A)** Scheme of a general approach to assess gut microbial, both bacteria and viruses, communities of wild-harvested *Ciona*, as described in (84). Briefly, after dissection, gut is homogenized, centrifuged at slow speed to eliminate host tissue/debris and then 0.22 µm filtered to isolate the bacterial fraction (bound to the filter membrane) and viral fraction (present in the flowthrough). Both samples are then processed for extraction of DNA and sequencing. **(B)** Bacterial relative abundances classified to phylum, with Proteobacteria split by class, and only phyla containing at least 1% of reads across all samples are designed by color. Phylogram on left denotes similarity of samples. SD, San Diego samples; N, Naples samples; WH, Woods Hole samples, (a) starved animals, (b, c) two sets of animals not starved. Reprinted from (85). **(C)** Taxonomy and abundance of all dsDNA viral contigs from trisected gut samples. Magenta rectangles highlight the most abundant viruses, the Caudovirales. SC, stomach cleared (starved animals); SF, stomach full; MC, midgut cleared (starved animals); MF, midgut full; HC, hindgut cleared (starved animals); HF, hindgut full. Reprinted from (84).

Studies have been focused on both describing, through sequencing, the diversity of the microbial communities present, i.e., bacteria and viruses, in the *Ciona* gut (**Figure 4A**) (84, 85), and in isolating and culturing a diverse assortment of microbes and viruses, i.e., bacteria, bacteriophages, and fungi (Dishaw et al. unpublished (62);). Amplicon sequencing of 16S rRNA genes (V4 region; Illumina MiSeq) from *Ciona* whole gut microbiota, using individuals either starved or not starved, revealed the likely presence of a core bacterial community, consisting of 35 operational taxonomic units (OTUs) shared among animals collected in disparate geographic areas, e.g., San Diego (California, U S A) and Naples (Italy) (85). This core microbiota includes (ordered by abundance): Gram-negative bacteria of Proteobacteria, Bacteroidetes, Fusobacteria, and Verrucomicrobia phyla, and Gram-positive bacteria of Firmicutes and Actinobacteria phyla; however, the abundance of individual core OTUs can vary considerably (**Figure 4B**) (85). Interestingly, these are the same phyla that appear to be most important among many other animals, including humans. The preservation of distinct microbial taxa in the gut of a filter-feeder highlights the likely presence of strong selective forces shaping these communities. Recently, based on morphological and developmental characteristics along with analysis of mitochondrial genome features, i.e., gene order, number and size of noncoding regions, compositional features, and sequence divergence (86), it was recognized that the two cryptic species of *Ciona intestinalis*, previously named types “A” and “B” (87), have been in fact diverging and resulting in two distinct species, now recognized as *Ciona robusta* and *Ciona intestinalis*, respectively (88). Also, it is important to mention that the original draft genome and the subsequent analysis of immunity were performed on *Ciona robusta*, though at the time recognized as *C. intestinalis* (23, 89). Analysis of the *Ciona* gut microbiota reveals that the stably associated core microbial community is shared among both species, which includes animals from the North Atlantic population (represented by the Woods Hole, Cape Cod, Massachusetts, USA) (85), suggesting that these microbes are essential to the *Ciona* genus. However, some unique taxa between the two species may also suggest, consistent with phylosymbiosis theory, that in *Ciona* some interplay exists between species divergence and microbiomes (82, 83).

The composition of animal-associated bacterial communities can be affected by a variety of exogenous components and includes their viruses, known as bacteriophages or phages. The characterization of the *Ciona* gut virome, from animals collected in San Diego (California, USA) over two separate years, has demonstrated that i) the viral community of the gastrointestinal tract is distinct from the surrounding seawater, ii) some temporal variability exists, and iii) phages predominate the viral communities. The top 10 viral contigs share sequence identity with phages infecting described members of the *Ciona* core microbiota, including *Flavobacteria*, *Shewanella*, *Pseudoalteromonas*, and *Vibrio*. Further, most of the phages appear to be temperate phages, which most often exist as prophages stably integrated within bacterial genomes. Interestingly, animals that had their guts cleared, i.e., the starvation group, revealed significantly more unique viral sequences (84). This suggests that starvation is stressful on the gut microbiome and this process induces prophage release from lysogenized bacteria. Finally, compartmentalization of viruses was noted in the digestive tract, with the midgut possessing the largest number of unique viral sequences (**Figure 4C**) (84). These findings reaffirm the importance of studying the role of phages in shaping the structure of the *Ciona* gut microbiome likely involving both lytic infections and lysogenization of bacteria.

## Interactions Between Different Components of *Ciona* Gut Environment

Colonization by diverse types of beneficial and commensal microorganisms, with the exclusion of those that are pathogenic, and the regulation of the spatial organization of microbiota along the digestive tract are determined by complex interactions among many factors, both endogenous and exogenous, within the gut environment. This complexity is discussed below, although it will not include a further examination of factors that are of host origin and also involved in this process, such as: 1) chemical features, e.g., pH, that can be determined by the host but also influenced by microbes (90, 91), and 2) biophysical forces, e.g., gut motility, that are driven by the enteric nervous system and also influenced by the environment; both help shape gut microbiota composition (92).

### Mucus Layer and Microbiota, a Dynamic Interplay

Beyond a protective role in preventing damage to the intestinal epithelium caused by food and digestive enzymes (93), an additional function of the mucus layer is to provide a niche for microbial colonization (38, 94–96), which can indirectly protect against overgrowth and/or attack from invading pathogens. As first introduced above, in mammals, a set of large glycoproteins, the MUCs, provide mucus with its viscous properties; they can be either secreted to form a gel-like structure or be produced as transmembrane molecules that help to form the epithelial glycocalyx (36, 97). Around 70–80% of the mass of MUCs is O-glycosylated, with N-acetyl-galactosamine, N-acetyl-glucosamine, galactose, and fucose serving as the main oligosaccharides attached to the protein core (94, 95, 98, 99). This differential glycosylation of MUCs is a means for selection, proliferation, and hence colonization by microbes within the diverse regions of the digestive tract. Indeed, the capacity to adhere to, and often consume, the mucus layer within the gastrointestinal tract serves as the first layer of selection in the regulation establishment of the microbiota (95, 96). Region-specific glycosylation of MUCs along the gastrointestinal tract has been observed in humans and rodents (100, 101), supporting a role for the MUC O-glycans in the selection of the gut microbiota. The preferential binding sites of O-glycans act as ligands for bacterial adhesins, and as carbon and energy sources to the surrounding microbial community. To attach and interact with mucus, symbiotic bacteria can either use mucus-binding proteins or extracellular appendages such as flagella, pili, and fimbriae (95, 96). Some intestinal bacteria secrete enzymes such as glucosidases that facilitate the degradation of MUC oligosaccharides, which can be further utilized/metabolized by other resident microorganisms (97, 102, 103); synergism in carbon-utilization strategies is common in polymicrobial communities. Collaborative strategies are beneficial in the utilization of mucus, since a combination of diverse enzymatic activities is required (104, 105). In mammals, for example, mucolytic bacteria include species as diverse as *Akkermansia muciniphila*, *Bacteroides thetaiotaomicron*, *Bifidobacterium bifidum*, *Bactetroides fragilis*, and *Ruminococcus torques* (96, 102, 106). Conversely, pathogens or pathobionts often use antagonistic strategies to compete with commensal microflora for access to nutrients; thus, availability of carbon sources can influence pathogens and commensal colonization success and their niche adaptation (107, 108).

Another function of gut MUCs is to influence the formation of mucosal biofilms, which are bacteria organized into complex surface-attached communities (109, 110); biofilms confer a variety of advantages as opposed to a planktonic existence. Studies of natural biofilms within intestinal mucus are difficult for various reasons, including the rapid turnover of mucus, i.e., collecting shed mucus could be informative, and a lack of proper preservation techniques. However, *in vitro* assays reveal that MUCs significantly influence biofilm formation among different strains of bacteria (111–113). In *Ciona*, a gene product related to human gel-forming MUCs, Ci-GFM1, exists but its function and interaction with gut microbes are not yet known; however, future studies of these molecules could help reveal essential processes of colonization. More attention has been devoted instead to another component of *Ciona* mucus, chitin. The chitin-rich mucus may serve important structural or physical purposes; a well-known example is the peritrophic matrix of the insect midgut, which, among several functions, helps mediate protection against parasitic infections (56, 114).

**Figure 5:**
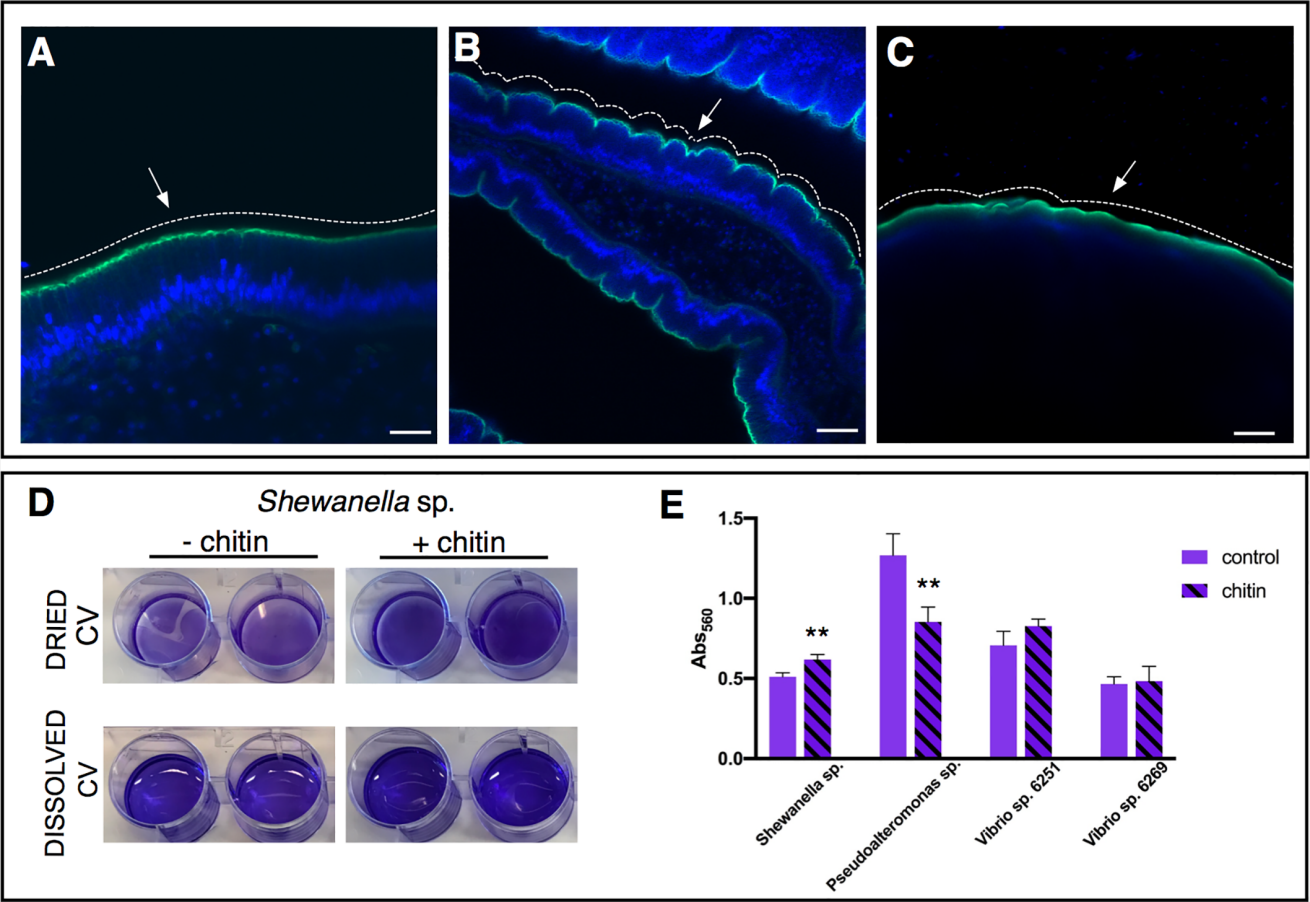
Role of chitin-rich mucus in *Ciona* gut. **(A–C)** Chitin enhances gut epithelial barriers as revealed by DSS-induced colitis-like phenotype. **(A)** The smooth continuous layer seen in control stomach epithelium (arrow) appears as having furrow structures **(B)** in the stomach of DSS-treated animals (arrow). **(C)** The surface of the epithelium remains mostly smooth (arrow) when treatment with DSS occurs in presence of chitin microparticles. Alexa Fluor 488-Phalloidin, green; DAPI, blue. Asterisk, gut lumen; white dotted lines highlight the surface morphology of the epithelium, smooth or with furrows. **(A–C)** reprinted from (47). **(D, E)** Biofilm assays using bacterial isolates cultured from *Ciona* gut are performed *in vitro*, with or without hydrolyzed chitin. **(D)** *Shewanella* sp. biofilms grown for 2 days in the presence/absence of chitin are quantified by crystal violet (CV) staining; plate shown with dried or solubilized stain (in acetic acid). A more intense staining is observed in presence of chitin. **(E)** Quantification of the solubilized CV staining of biofilms, measured with a microplate reader at OD_560_, of various bacterial isolates from the *Ciona* gut, with or without hydrolyzed chitin present. *Shewanella* sp. and *Pseudoalteronomas* sp. biofilm formation is influenced by chitin, whereas no effect was noted in two different *Vibrio* spp. isolates. Asterisks indicate statistical significance calculated using two-tailed *t*-tests (P<0.01). Scale bar: **(A)**, 20 µm; **(B)**, 40 µm; **(C)** 100 µm.

Mammals, including humans, lack chitin synthase genes and thus cannot synthesize chitin oligosaccharide chains; instead, chitin sources can only be acquired *via* diet and can include feeding on arthropods and fungi (115). Various studies of chitin particles and its derivatives, such as chitosan (deacetylated form of chitin), report its role as a prebiotic, alleviating metabolic disorders and modulating the composition of gut microbiota (116, 117), and as an immune modulator (118). This effect of chitin particles in regulating gut microbiota and immune responses has also been noted in fish aquaculture (119, 120). In *Ciona*, chitin-rich mucus is endogenously produced and exuded as part of the gel layering on the epithelial surface; it also acts as part of a mucus-net that entraps and encases food particles, a process that begins in the pharynx (46). Functionally, disruption of these fibers allows direct microbial contact with gut enterocytes, suggesting an important role as a physical barrier (50). For example, treatment with dextran sulfate sodium (DSS), a compound employed to induce experimental colitis-like phenotypes in mammals (121, 122) by mediating biophysical changes to the structure of mucus, contributes to bacterial colonization of the inner mucus layer and results in inflammatory responses (123, 124). This treatment also induces a similar colitis-like phenotype in *Ciona* by disrupting the chitin-rich mucus layers and inducing inflammatory responses (47). However, exposure to exogenous chitin microparticles enhances the physical barriers of the intestinal epithelium and attenuates the colitis-like features and inflammation, demonstrating a strong protective function of chitin in *Ciona* (**Figures 5A–C**) (47); a related protective effect of exogenous chitin has also been observed in the mouse model, where chitin is not endogenously produced (125). Moreover, chitin can be utilized as a carbon source and often in the formation of biofilms, as has been observed in *Vibrio cholerae*, a facultative human pathogen (126, 127). Gut microbes isolated from *Ciona*, such as *Shewanella* sp. and *Pseudoalteromonas* sp., may be influenced by chitin-utilization pathways that also influence biofilm formation, likely shaping settlement outcomes (**Figures 5D, E**) (46).

### Innate Immune and Microbiota Crosstalk

**Figure 6:**
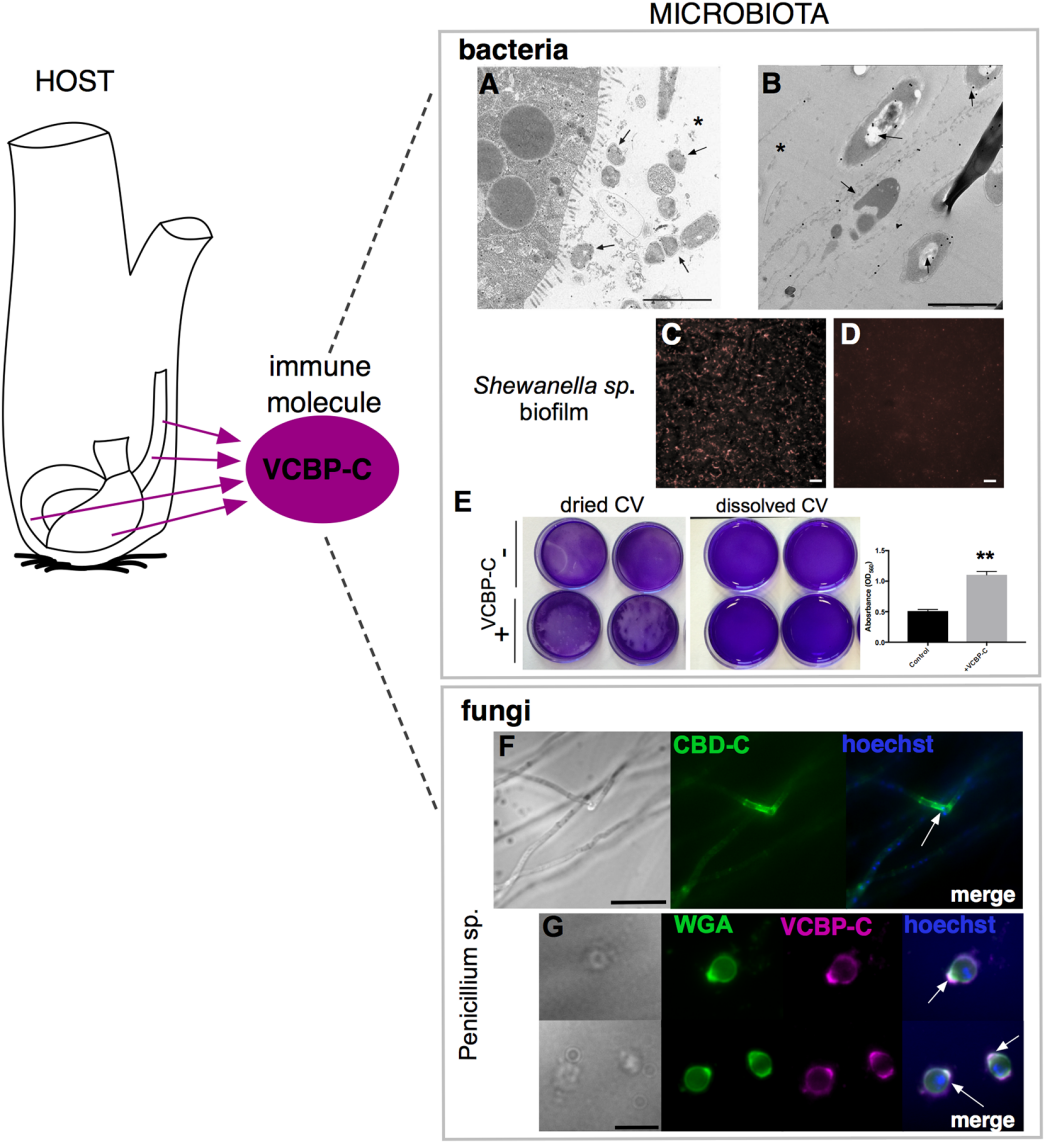
VCBP-C interaction with gut microorganisms. In *Ciona*, VCBP-C proteins are produced and secreted into the gut lumen by the epithelial cells of the digestive tract and are able to interact with different components of microbiota, i.e., bacteria and fungi. **(A, B)** Immunogold staining reveals VCBP-C binding to bacteria in the stomach; experimentally introduced bacteria, such as **(A)** *B cereus* and **(B)** *E coli*, are detected with anti-VCBP-C antibody in the lumen and in immediate proximity to stomach wall (arrows). Asterisk, stomach lumen. Reprinted from (48). **(C–E)** *Shewanella* sp. biofilms cultured in the presence of VCBP-C. Immunofluorescent staining, using anti-VCBP-C antibody and detected with Alexa Fluor 594 (red), reveals **(C)** VCBP-C bound to the bacteria grown as biofilm, whereas **(D)** no staining is observed in control bacterial biofilm grown without VCBP-C. Reprinted from (46). **(E)** *Shewanella* sp. biofilm grown for 4–5 days in the presence/absence of VCBP-C quantified by crystal violet (CV) staining; plate shown with dried (left) or solubilized stain (in acetic acid; right images). The graphic on the right shows quantification of CV staining, measured with a microplate reader at OD_560_, and highlights an increase in biofilm formation in presence of VCBP-C. Asterisks indicate statistical significance calculated using two-tailed *t*-tests (P<0.01). **(F)** Immunofluorescence with recombinant IgG1-Fc-CBD-C probe on whole *Penicillium* sp. fungi grown in liquid medium reveals binding of the CBD (green) probe to chitin molecules localized in specific regions of the fungal hyphae (arrow). **(G)** Immunofluorescence with anti-VCBP-C antibody on fungal spores isolated from liquid culture of *Penicillium* sp. and incubated with recombinant VCBP-C protein reveals binding of VCBP-C (green) on chitin molecules localized in specific regions of spore surface (e.g., bud scars, arrows). VCBP-C specific binding to chitin molecules is confirmed by its co-localization with Wheat Germ Agglutinin (WGA) staining (magenta), which is a lectin known to recognize chitin on fungal surfaces. Reprinted from (62). Scale bars: **(A)**, 2 µm; **(B)**, 1 µm; **(C, D)**, 20 µm; **(F)** 25 µm; **(G)** 10 µm.

Secreted effectors of the immune system, such as antimicrobial peptides (AMPs) and IgA in vertebrates, are other important components of mucosal immunity that integrate into the gut mucus layers and shape the ecology of the microbiome while fortifying barrier defenses. The discovery and characterization of the VCBPs, with their peculiar protein structure (IgV domains and CBD) and production by both blood cells and by the gut epithelium, have made them especially attractive for studies of immune interaction with microbes. So far, among the VCBP molecules, VCBP-C has been most characterized, revealing its interplay with distinct components of the gut environment. Although there is not a clear demonstration of the specific function of each VCBP-C domain, it has been observed that this protein co-localizes with bacteria in the *Ciona* gut mucus (46) and can bind both Gram-positive (*Bacillus cereus*) and Gram-negative (*Escherichia coli*) bacteria, *via* its first Ig domains (**Figures 6A, B**) (48). It has been demonstrated that these domains are also responsible for the opsonic activity of the protein, binding and presenting bacteria to be recognized by granular amoebocytes, thus increasing bacterial clearance *via* phagocytosis (48). Additionally, the CBD of VCBP-C can bind to chitin present in the mucus, where its colocalization has been observed since the early stages of development, specifically within the developing intestine (**Figures 3E–I**) (46). Recently, it has been demonstrated that the CBD also binds chitin molecules present in the cell wall, sporangia, and spores of fungi isolated from the *Ciona* gut (**Figures 6F, G**) (62). Hence, VCBP-C, to our knowledge, is the first secreted Ig-containing immune effector with the capacity to directly promote transkingdom interactions by simultaneously binding diverse gut microbial components; this promiscuity may have broad implications in modulating the establishment, succession, and homeostasis of gut microbiomes (62). Future studies for deciphering the significance of these interactions and the implication for host health are necessary.

Whereas the functional consequences of VCBP-C in bridging interactions between bacteria and fungi remain under investigation, *in vitro* experiments have demonstrated a role for VCBP-C in directly modulating the formation of bacterial biofilms (**Figures 6C–E**). Upregulation of some biofilms has been noted, for example, in *Bacillus* sp., *Shewanella* sp., and *Pseudoalteromonas* sp. isolated from the *Ciona* gut; downregulation or no effects have been observed with other bacterial species, while some *Vibrio* species, for example, seem more affected by the presence of hydrolyzed chitin (46, 128). This VCBP-C-specific activity is concentration-dependent and relies not only on the Ig domains but also on the CBD, whose presence appears to serve an important role (46). This differential influence on biofilm formation among distinct bacterial taxa remains to be explored further.

Although speculative, analogies from a functional point of view can be drawn between VCBP-C and mucosal antibodies from other animals, e.g., IgA in mammals or IgT in fish. In mammals, IgA acts as a component of barrier defenses, regulating bacterial adherence to the epithelial surface *via* immune exclusion processes that include agglutination and/or opsonization of bacteria (129, 130). Moreover, IgA may also influence non-pathogenic bacterial biofilm formation (131–134). In an analogous manner, VCBP-C may modulate adherence and biofilm formation on *Ciona* gut epithelial surfaces, where the settlement of some transient bacteria is regulated in a process that resembles immune exclusion by IgA while serving other important roles in maintaining gut homeostasis (46). Likewise, in a *Ciona* DSS-induced colitis-like model, VCBP-C expression in the gut was found to be up-regulated, likely for defensive purposes, further supporting a role analogous to that of IgA in mammals, where it recognizes commensal bacteria that preferentially affect inflammatory bowel disease (IBD) susceptibility (47, 135, 136). A related role for VCBP-C in modulating dysbiosis is predicted also during animal development (metamorphosis stages), when the gut microbiome is being established. Indeed, juveniles experimentally fed Gram-positive or Gram-negative bacteria revealed a distinct up-regulation of VCBPs. Specifically, *B. cereus* and *E. coli* increase mRNA level of VCBP-A and VCBP-C, respectively (49).

As briefly mentioned above, the *Ciona* gut also expresses TLRs, and this likely contributes to not just an innate recognition of microbial products but helps establish and/or maintain a bidirectional regulation of the microbiome. In mammals, distinct TLRs recognize different microbial ligands that often form conserved shapes or patterns, for example, lipopolysaccharide from Gram-negative bacteria. In *Ciona*, on the contrary, multiple ligands are recognized by the two TLRs (65) and this promiscuity likely evolved to counter the effects of a reduced assortment of available receptors. These ligands include zymosan, heat-killed *Legionella pneumophila*, double-stranded RNA (PolyI:C), and flagellin. Their binding to CiTLRs induces the transcriptional activation by NF-kB, and additionally, Poly(I:C) and flagellin trigger *tumor necrosis factor α* (*CiTNFα*) expression (65). A slight upregulation of *CiTLRs* was noted in the DSS mediated colitis-like phenotypes (47). However, other functions of CiTLRs within the *Ciona* gut, and specifically in regulating or mediating host-microbial symbiosis, require further investigation.

## Multi-Omics Approaches for Studying Host-Microbial Interactions

The development of high-throughput -omics technologies, and their current acceptable costs, has accelerated a new era of opportunity in microbiome studies defined by the ability to evaluate most aspects of the community, without the limitations imposed by culturing (137) or targeted approaches, thus producing a high volume of data representing genes, transcripts, proteins, and metabolites (138). The associated multi-omics technologies include shotgun metagenomics and metatranscriptomics, along with mass spectrometry-based metaproteomics and metabolomics. A specific goal of broad sequence-based technologies, such as metagenomics, is to provide a glimpse of the total genetic content, enabling the elucidation of the composition and the functional potential of the whole community (137, 139). Total genetic content also facilitates prediction of the theoretical transcriptome; however, *via* metatranscriptomics or RNA sequencing (RNA-seq), a process involving high-throughput sequencing of RNA from the entire microbial community, a true representation of gene expression can be evaluated rather than predicted from assembled genes (140). Metatranscriptomics can also be used to sample host tissue gene expression. These transcriptome data, thus, facilitate the characterization of functional changes across different contexts, in support of the inference of how microbiome interactions (i.e., host-microbe and microbe-microbe) regulate community activities (141). However, due to inherent limits of the data resolution and interpretations, metagenomic and metatranscriptomic analyses alone are not sufficient for drawing conclusions of the actual biological mechanisms within a community. Hence, metaproteomics and metabolomics, mass spectrometry-based methodologies, can serve as complementary approaches to approximate the actual phenotypes by studying the protein content of microbial communities (142) or small-molecule metabolites within a given biological sample (143), respectively, offering a snapshot of the global physiological state of the community. Thus, integrated multi-omics analyses of the gastrointestinal microbiome provide a comprehensive foundation for hypothesis-driven studies of host-microbe interactions.

### Multi-omics Approaches for Studying *Ciona* Gut Environment in Physiologic Conditions

**Figure 7:**
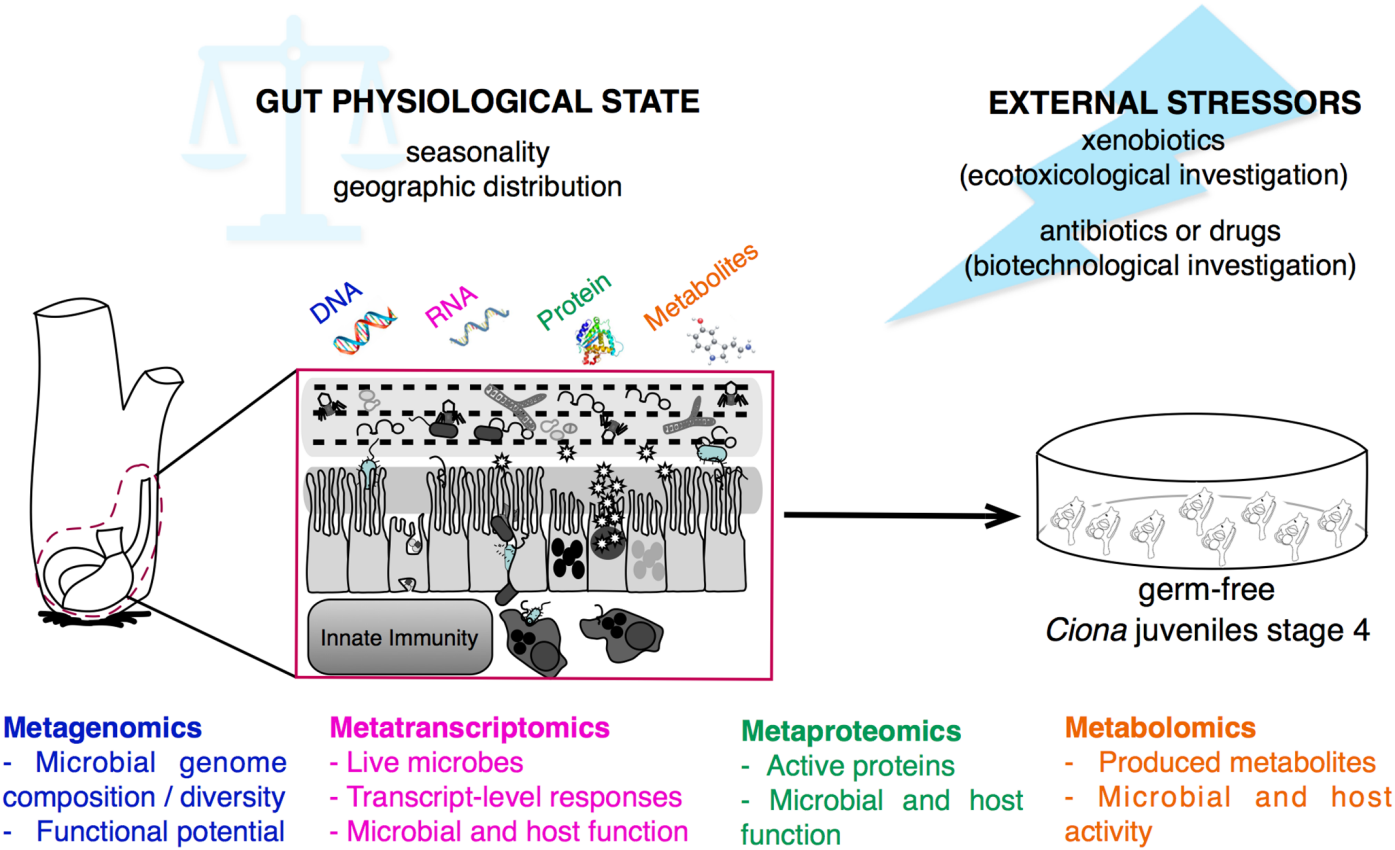
Multi-omics approaches for studying gut-microbial interactions using the model organism *Ciona robusta*. Schematic representation of the metagenomic, metatranscriptomic, metaproteomic, and metabolomic approaches that can be performed on *Ciona* digestive tract, investigating the physiological state, and homeostatic condition, of host-microbial interaction during different seasons and geographic locations, or after experimental treatments. This holistic view of the microbiome composition can be impacted by external stressors, such as environmental factors, i.e., xenobiotics, or biomedical treatments, i.e., antibiotics or drugs. Hypotheses derived from host-microbial interactions leveraging multi-omics approaches can be tested and validated *in vivo* using germ-free *Ciona* juveniles.

In *Ciona*, the studies of host-microbe interactions within the gut have thus far been focused on the identification and/or isolation of specific strains from the gut environment. Bacterial and viral components were first characterized *via* 16S rRNA amplicon and viral metagenomic sequencing, respectively, revealing a stably associated microbial community shared by both species, *Ciona robusta* and *Ciona intestinalis* (85), and a viral community predominated by bacteriophages (84, 144). Simultaneous investigations using molecular, cellular, biochemical, and microbiological techniques identified a role for the interaction between host immune effectors, i.e., VCBPs, and the microorganisms present in the digestive tract (46, 59, 62, 145). However, to expand the rate at which discovery is made, these studies will benefit from leveraging -omics approaches. For example, metagenomic and metatranscriptomic sequencing of the *Ciona* gut, either in its entirety or sub-divided into stomach, midgut, and hindgut, may help to identify the dynamic interactions between host and microbiome that help define factors shaping homeostasis among different seasons and distinct geographic locations. Moreover, metaproteomic and metabolomics may help identify active proteins, enzymatic pathways, and metabolites used for mediating interactions between host and microbes within the gut; recent examples focus on the tunic of three ascidian species, including *Ciona* sp. (146, 147) (**Figure 7**). Subsequently derived hypotheses can be tested *via* experimental approaches, if isolation of the identified components is possible, and combined into functional assays. Indeed, these experiments in *Ciona robusta* are now augmented with important tools, such as the ability to rear short-term *germ-free* juveniles, essential in studies of host-microbial interactions (148) (**Figure 7**). Colonization of germ-free animals, for example, could help facilitate studies aimed at understanding if colonization of the gut shapes developmental outcome, since animals like *Ciona* sp. complete development while exposed to the environment.

Likewise, leveraging simple *in vitro* assays, such as microbial biofilm assays, facilitates studies of host effectors (and other isolated factors) with individual components of the microbiome (128); data from these experiments can help pose hypotheses to be tested *in vivo.* Hence, the use of multi-omics approaches in *Ciona*, as well as in other organisms, may enable a more holistic view of microbiome composition and function at multiple layers (141, 149), facilitating hypothesis-driven experimentation to ascertain mechanisms of homeostasis and help define roles for immune effectors, and their interactions with specific components of these complex microbial communities.

### Multi-Omics Approaches in *Ciona* Model for Studies in Ecotoxicology and Biotechnology

Research on host-microbial interactions are mostly focused on revealing the significance to host physiology, homeostasis, disease, health, and fitness (150), whereas toxicology studies investigate the effects of chemical compounds on organisms, examining the accumulation, biotransformation, elimination, and effect in tissues (151). However, the epithelium and its associated microbiota lie at the interface between host and its environment, serving as a first line of defense against contaminants and environment stressors. In a recent mini-review, *Marie B.* and coworkers advocate for the development of a “microbiome-aware ecotoxicology,” emphasizing the importance of investigating host-microbiome interactions in light of ecotoxicological implications, while discussing important conceptual and technical pitfalls associated with study design and interpretation (151). To date, most studies have focused on the effects of environmental contaminants, such as pesticides, antibiotics, heavy metals, nanoparticles, microplastics, or compounds with endocrine-disrupting activity on gut microbiota (17, 152–154), mostly describing changes to the composition of bacterial communities *via* 16s rRNA sequencing (155–157). Whereas, fewer studies have focused on the functional capability of the gut microbiota in metabolizing a wide range of these xenobiotics (152) or in the production of secondary metabolites that may mediate toxicity to the host (158). Additionally, most of these studies have been performed in mammals; however, since most of the contaminants are released in aquatic environments, an important consideration is how aquatic organisms, which face continuous exposures, are impacted (17). A recent ecotoxicological investigation leveraged *Ciona robusta* as a model, assessing the impact of the combined effect of both industrial pollution (from the industrial area of Bagnoli-Coroglio, Naples, Italy) and two temporal patterns of turbulence events on the gut environment (159). Four broad categories were investigated: oxidative stress, innate immunity, host-microbial interactions, and integrity of gut epithelial barrier, with a focus on expression patterns of oxidative pathway and immune response genes (159). In particular, evidence for the induction of antioxidant defenses was detected after 7 days of exposure to static, polluted, sediment, with higher levels of glutathione S-transferase and metallothionein gene expression observed. Mechanisms of adaptation to chronic exposure to chemical mixtures was also suggested, revealing that turbulence events inducing polluted sediment suspension and a consequent release of heavy metals and polycyclic aromatic hydrocarbons do not correlate with an antioxidant response in *Ciona*, but instead with a pro-inflammatory response, as suggested by the increase in gene expression of *CiTNFα*. On the contrary, the absence of effects on gene expression of molecules involved in the establishment and maintenance of barrier defenses, such as MUC, chitin synthases, and also VCBPs, suggested that the mixture of pollutants did not interfere with the host-microorganisms interaction in the gut (159). However, the availability of a more comprehensive multi-omics approach would further help elucidate, utilizing high throughput and untargeted methods, the impact of environmental pollutants on the composition, behavior, or metabolic potential of gut microbial communities as well as to the predictive interpretations of the functional impacts on the animal host (155).

Invasive species, such as filter-feeders like *Ciona robusta*, despite being constantly exposed to potential toxins in aquatic environments, often can adapt. For example, they possess adaptable “xenobiotic receptors” and antioxidant defense systems that help regulate their physiological responses to repeated exposures of potentially toxic compounds or other environmental stressors, such as temperature fluctuations (160, 161). Also, as a tunicate, *Ciona* occupies an unusual ecological and evolutionary position, since it is both an invertebrate and part of a group that is a sister clade to the vertebrates (162). As such, it can complement ecotoxicological studies that leverage other filter-feeding organisms, such as bivalves (which are more distantly related to vertebrates), more often used for marine environmental monitoring (160). Thus, *Ciona* can be an informative system for understanding how animals respond to or tolerate certain chemicals that are foreign to the body, i.e., xenobiotics. This adaptability also relates to the gut microbiome, which may help modify such compounds in ways that protect or promote the survival of the host; indeed, this can serve as a strong selective force favoring bacteria that can tolerate such exposures and even metabolize the compounds. Therefore, detecting changes to the structure of gut bacterial communities may serve as important bioindicators, while also providing opportunities to predict community-level responses to short- and long-term exposures to environmental pollutants. These observations may help us better understand the capacity of the microbiota to both transform specific environmental pollutants and help adapt the host to contaminated environments (17) (**Figure 7**).

Furthermore, because the gut microbiota is so metabolically active, it has been regarded as an “invisible organ” that may directly or indirectly modulate the function of drugs. The direct effect includes the biotransformation of drugs or their metabolites into products with altered bioactivities, thus changing their efficacy; whereas, the indirect effect involves more complex host-microbial interactions that affect host pathways for xenobiotic metabolism or transport (163, 164). It is now also evident that the microbiota can help mediate the effect of some drugs that target the immune system, and that changes to the structure or function of the microbiome represent an unanticipated limitation of the treatment (163). These data support the use of *Ciona robusta* as a model organism for investigating the impact of natural products/drugs on innate immune responses, and for studies of the role of the microbiota in metabolizing drugs and other dietary compounds that may explain observed variation in drug pharmacokinetics (165, 166). These data emphasize the importance of also focusing pharmacogenomics studies on genes encoded by the gut microbiota (163). For the reasons described above, *Ciona robusta* is particularly suited for studies of host-microbial interactions and can be further leveraged for investigating a role for the gut microbiome in drug metabolism, *via in vivo* and *in vitro* approaches, and multi-omics technologies, providing a comprehensive overview of the microbiome’s metabolic impact and function (**Figure 7**). Indeed, future drug discovery efforts depend on harnessing powerful new technologies and model systems, while integrating information from sequenced genomes, functional genomics, protein profiling, metabolomics, and bioinformatics, in a manner that ensures a comprehensive systems-based analysis that furthers our understanding of the complexities of health and disease (167, 168).

## Conclusion On the Use of *Ciona Robusta* in Studies of Host-Microbial Interactions

The modern realization that complex animal-associated microbial communities most often serve as vital symbiotic interactions or evolved interdependencies shaping physiology and homeostasis has resulted in an urgent need for studying diverse model systems. Here, we focus our attention on the invertebrate chordate model *Ciona robusta*, first proposed by Dishaw et al. in 2012 as a novel tractable model system for studies of host-microbial interactions within the gut (18), and review the important advancements made thus far. These include i) a description of the components of the gut environment from host side (i.e., structure and organization of the mucus layers, along with immune molecules produced by the epithelium) (46, 48–50), ii) characterization of the microbiota (i.e., composition of bacteria and viruses) (62, 84, 85, 144), and iii) the functional characterization of some of the interactions between these various elements (i.e., mucus layer-microbiota and microbiota-innate immunity) (46–48, 62). As mentioned throughout this review, due to both its phylogenetic position, anatomic features, and reduced and well-known genome, *Ciona robusta* is a valuable model organism for such studies as it represents a link between the invertebrate and vertebrate lineages and can elucidate conserved as well as novel mechanisms that shape gut homeostasis. Moreover, as a filter-feeding organism that concentrates carbon sources and other compounds, it is also a valid model for both ecotoxicological studies and the impact of environmental pollutants on gut ecology; it is also a relevant resource for studies of biomedical relevance, e.g., how certain compounds impact the gut microbiome, host immunity, or the chemistry or biophysics of the gut lumen.

Although some aspects of the gut ecosystem have now been explored and described in *Ciona*, e.g., mucus-microbiota and immune-microbiota, continuing to advance the field will require and benefit from expanded genetic approaches as well as the integration of more advanced multi-omics technologies that will help comprehensively define the system without the bias of approaches targeting specific genes or gene products. Results from these studies will increase our understanding of the basic mechanisms of host-microbial interactions leveraging more holistic views of the forces shaping the gut both as an ecosystem and an organ, whose function is expanded by the microbiome (141). This will finally help us move beyond simply describing “who is there” in the gut to unraveling “what they are doing,” which is the ultimate goal of such studies. Although much progress has been made, a lot remains to be done, and model organisms such as *Ciona robusta* can continue to contribute advancements in our knowledge, while helping to expand the perspectives from which we investigate complex symbiotic interactions that shape animal physiology and, ultimately, health and disease.

## Author Contributions

AL and LJD conceived the review and drafted the first full version of the manuscript. ON, CGFA, and PS contributed ideas and edited and revised later versions of the manuscript. All authors contributed to improving the manuscript and have approved the submitted version.

## Funding

This work was supported in part by grants from the National Science Foundation (IOS1456301 and MCB1817308), Johns Hopkins All Children’s Hospital Foundation Research Grant, and a USF College of Medicine Internal Award to LJD, and “Sperimentazioni pilota finalizzate al restauro ambientale e balneabilità del SIN Bagnoli-Coroglio (ABBaCo)” funded by the Italian Ministry for Education, University and Research (grant no. 508 C62F16000170001) and “Antitumor drugs and vaccines from the sea (ADViSE)” funded by the Regione Campania POR Campania FESR 2014/2020 Asse I. to AL and PS. The funders had no role in study design, data collection and analysis, decision to publish, or preparation of the manuscript.

## Conflict of Interest

The authors declare that the research was conducted in the absence of any commercial or financial relationships that could be construed as a potential conflict of interest.
